# Commissioning of a Versa HD^TM^ linear accelerator for three commercial treatment planning systems

**DOI:** 10.1002/acm2.13180

**Published:** 2021-02-22

**Authors:** Wolfram U. Laub, Brandon Merz, Monica Kishore

**Affiliations:** ^1^ Associates in Medical Physics (AMP) Greenbelt MD USA; ^2^ Department of Radiation Medicine Oregon Health and Science University Portland OR USA

**Keywords:** beam modeling, Eclipse, linear accelerator commissioning, Monaco, Pinnacle, Versa HD

## Abstract

In a mixed‐vendor radiation oncology environment, it is advantageous if the department's treatment planning system (TPS) supports the linear accelerators of different vendors. In this publication beam data collection and modeling for the Versa HD linear accelerator in Monaco, Pinnacle, and Eclipse are discussed. In each TPS static field, Intensity‐Modulated Radiation Therapy (IMRT) step and shoot, and Volumetric‐Modulated Arc Therapy (VMAT) plans for flattened and flattening‐filter free photon beams of all available energies were evaluated for field sizes >3 × 3. To compare passing rates, identical beam model validation plans were calculated in each TPS. Eclipse, Monaco, and Pinnacle beam models passed validation measurements in homogeneous materials for a variety of treatment fields, including static, IMRT, and VMAT. In the case of Eclipse, the “dosimetric leaf gap” parameter was found to be critical for passing rates of VMAT plans. The source size parameter plays an important role as well for small fields. In the case of Pinnacle the multileaf collimator offset table needed to be optimized for better VMAT QA results. Each of the investigated treatment planning systems met the criteria to be used clinically in conjunction with Elekta Versa HD linear accelerators. It can be of great advantage to have the option to operate a TPS and linear accelerator from different vendors, as decisions surrounding linear accelerator or TPS purchases are very complicated and not just limited to technical considerations.

## 1 INTRODUCTION

The decisions surrounding linear accelerator or treatment planning system (TPS) purchases are very complicated and not just limited to technical considerations. Ideally, the linear accelerator decision should neither drive nor force a decision to switch to a different TPS, as this could be very disruptive and costly for a radiation oncology department. On the other hand, the existing TPS should not force the decision to purchase a linear accelerator from a specific vendor. In a mixed‐vendor radiation oncology environment, it is therefore advantageous if the department’s TPS supports the linear accelerators of different vendors.

The Versa HD machine from Elekta has the possibility to use fattening‐filter free (FFF) beams, namely 6 and 10 MV‐FFF, and is equipped with the latest Agility 160 multileaf collimator (MLC). The Agility 160MLC has 160 motorized leaves arranged in 2 banks of 80. With this MLC, different techniques are supported, namely the delivery of conformal, static MLC field, Intensity‐Modulated Radiation Therapy (IMRT), and Volumetric‐Modulated Arc Therapy (VMAT) plans.

The Monaco (Elekta, Atlanta, GA) and Pinnacle (Philips, Fitchburg, WI) TPSs supported Versa HD linear accelerators (Elekta, Atlanata, GA) equipped with the Agility 160MLC and FFF beams soon after market clearance. Starting with TPS version 13.6 MR0.7, Eclipse (Varian, Palo Alto, CA) included the support of planning for the Elekta machine VersaHD with FFF beams as well. The only limitation being that while Eclipse and Pinnacle only support the IMRT step and shoot technique for Versa HD linear accelerators, Monaco also supports the dynamic MLC delivery of IMRT plans. This is not necessarily a major limitation, as it has been shown that dosimetrically the IMRT step and shoot technique and dynamic MLC delivery produce comparable results. The difference is that compared to dynamic MLC delivery the step and shoot technique can be between 15 and 50% slower, while the number of monitor units required is about less.^1^, ^2^

It is important to stress the comprehensive program required when acquiring both beam data for model generation as well as recording verification measurements.^3^ A model can only be tuned to the quality of its inputs. Similarly, any error in the calibration of the verification system will corrupt the tuning and thus quality of the beam model, regardless of the quality of the original beam data.

In this publication the beam data collection and modeling process for a Versa HD linear accelerator in Monaco, Pinnacle, and Eclipse are discussed. In all three TPSs static field plans, IMRT step and shoot plans, and VMAT plans for flattened and FFF photon beams were evaluated. To compare passing rates, identical beam model validation plans were calculated in all three TPSs.

## 2 MATERIALS AND METHODS

The commissioned Versa HD has five photon energies (6 MV, 6 FFF, 10 MV, 10 FFF, and 18 MV) and five electron energies (6, 8, 10, 12, and 15 MeV).

Beam modeling guides for Monaco, Pinnacle, and Eclipse were carefully reviewed. In an attempt to minimize the amount of data to be collected, data were measured such that it met the beam data requirements of several TPS.

### 2.A Equipment

To ensure highest quality of our linear accelerator commissioning data, we followed guidelines and recommendations as published in task group report 106 (TG‐106).^4^ Profiles and percent depth dose curves (PDDs) were measured with the microDiamond detector type 60019 and large field profiles (20 × 20 cm^2^ and larger) with the LA48 linear array (both PTW‐Freiburg). The microDiamond has a high spatial resolution, which is critical for correct penumbra modeling of photon and electron beams.^5^ Output factors (Total‐Scatter Correction Factor [TSCF]) were measured with the microDiamond detector in water and collimator factors (Sc) with a 0.125 cc Semiflex detector type 31010 with a brass buildup cap in air (both PTW‐Freiburg). Absolute dose calibration was done with a 0.6 cc Farmer detector type 30013 for photons and an Advanced Markus chamber type 34045 for electrons (both PTW‐Freiburg). All scans were completed in an MP3‐M water phantom (PTW‐Freiburg).

Absolute output calibrations were verified independently by two physicists and through external OSLD testing (IROC Houston QA Center).

### 2.B Photon beam data collection

Profiles and PDDs were measured at source‐surface distance (SSD) 90 cm and profile depths were dmax, 5 cm, 10, 20, and 30 cm depths. For open fields, field sizes were (in cm^2^): 1 × 1, 2 × 2, 3 × 3, 4 × 4, 5 × 5, 7 × 7, 10 × 10, 12 × 12, 15 × 15, 20 × 20, 25 × 25, 30 × 30, 34 × 34 (this is the largest field size with MLCs open in the corners), 40 × 40 (this field has rounded corners), 20 × 5, and 5 × 20. Diagonal profiles were measured for a 40 × 40 cm^2^ field size at dmax, 5, 10, 20, and 30 cm depth. For wedged fields, measured field sizes were (in cm^2^): 2 × 2, 3 × 3, 5 × 5, 10 × 10, 15 × 15, 20 × 20, and 40 × 30 (this is the largest field size for wedged fields). At the time of commissioning, Monaco also required star scans at 10 cm depth for the largest wedged field every 10 degrees and for the diagonals across the 40 × 30 field. These measurements are no longer required according to the latest Monaco beam modeling guide.

Output factors (TSCF) were measured at SSD 90 and 10 cm depth (reference point depth) in water for the same field sizes as profiles and PDDs. Pinnacle did not require any additional collimator factor measurements. For Monaco it was necessary to take additional collimator factor measurements for all field sizes as listed in Table 1. For Eclipse it was necessary to measure a large number of additional output factors (TSCF) for each energy and for wedged and open fields as listed in Table 2. An important caveat for Eclipse is that while the beam model fitting will disregard output factors for field sizes 2 × 2 and below, these factors will still be used by Eclipse during the calculation of small fields. It is thus important to include these factors. Without small field output factors, Eclipse will calculate small fields with extrapolated values instead of actual acquired data and will give a warning message. Also worth considering is the effects of the location of the MLC and its dual role as a collimator in the X‐direction. The Eclipse multisource model is suited to model this.^6^

**TABLE 1:** Required collimator factor measurements for Monaco (for wedged fields collimator factors are only needed for square fields).

5 × 5	3 × 40	40 × 30
10 × 10	5 × 40	40 × 20
15 × 15	8 × 40	40 × 15
20 × 20	10 × 40	40 × 10
40 × 40	15 × 40	40 × 8
	20 × 40	40 × 5
	30 × 40	40 × 3

**TABLE 2:** Additional output factor (TSCF) data (per energy, wedged, and open fields) as required by Eclipse.

3 × 3	5 × 3	7 × 3	10 × 3	15 × 3	20 × 3	30 × 3	40 × 3
3 × 5	5 × 5	7 × 5	10 × 5	15 × 5	20 × 5	30 × 5	40 × 5
3 × 7	5 × 7	7 × 7	10 × 7	15 × 7	20 × 7	30 × 7	40 × 7
3 × 10	5 × 10	7 × 10	10 × 10	15 × 10	20 × 10	30 × 10	40 × 10
3 × 15	5 × 15	7 × 15	10 × 15	15 × 15	20 × 15	30 × 15	40 × 15
3 × 20	5 × 20	7 × 20	10 × 20	15 × 20	20 × 20	30 × 20	40 × 20
3 × 30	5 × 30	7 × 30	10 × 30	15 × 30	20 × 30	30 × 30	40 × 30
3 × 40	5 × 40	7 × 40	10 × 40	15 × 40	20 × 40	30 × 40	40 × 40

In addition, each TPS required description of calibration conditions (e.g., for SSD 100 cm @ 10 cm and a 10 × 10 field 100 MU = 67.5 cGy for a 6 MV beam). However, it is important that reference point conditions are always the same as the conditions under which the TSCF and collimator factors were measured. For this reason, the absolute dose value was also measured in water at isocenter point and at 10 cm depth (90 cm SSD) for reference field size is (e.g., for SSD 90 cm @ 10 cm and a 10 × 10 field 100 MU = 81.1 cGy for a 6 MV beam).

Each TPS has a different way of defining wedge factors. Pinnacle references everything back to an open 10 × 10 field (reference point). So for example, the wedge factor for a 3 × 3 wedged field is calculated as the ratio between the dose of a wedged 3 × 3 field relative to an open 10 × 10 field. Monaco requires input of the absolute dose of a wedged field under reference conditions at SSD 90 cm and then applies additional TSCF factors to wedged fields of different field sizes. Eclipse also requires input of the absolute dose of a wedged field under reference conditions at SSD 90 cm and a large number of TSCF factor measurements for wedged fields of different field sizes (Table 2).

MLC parameters are important for accurate small field or VMAT dose calculations.^7^, ^8^ Pinnacle’s MLC offset table defines small MLC offset values, which vary with the position of the MLCs in the field. This is a result of the curved Agility MLC tips and the fact that as MLCs travel across the field the angle of radiation incident on the leaf tip changes. As a result, the distance between the tip of the leaf and the point where beam has to travel through a thickness equivalent to the half value layer thickness of the beam, which is the MLC offset, changes as well. Recommended values can be found in the Pinnacle beam modeling guide, and should be manually adjusted up or down in increments of 0.25 mm (the size of one pixel of Elekta's portal‐imaging device, which is used for MLC calibration) to get highest VMAT QA pass rates. For Monaco so called ExpressQA measurements need to be completed on a 2D array or film. These measurements then allow Elekta's physics support to optimize MLC parameters during beam modeling. For Eclipse, dosimetric leaf gap (DLG) measurements can be acquired for each photon energy using plan files provided by Varian. Measurements were performed with a farmer chamber (FC‐65‐G) in a solidwater phantom at 95 cm SSD and 5 cm depth. Sweeping gap plans were imported into Mosaiq in DICOM format and delivered through the DICOM interface. Measurements were performed with sweeping gap distances of 2, 4, 6, 10, 14, and 16 mm. A spreadsheet was provided by Varian with instructions to calculate the MLC leaf gap parameter value for Eclipse for each energy from the resulting measurements.

### 2.C Electron beam data collection

Percent depth doses were measured for each electron energy at SSD 100, 110, and 120 cm for field sizes 3 × 3, 6 × 6, 10 × 10, 14 × 14, 20 × 20, 25 × 25, and 40 × 40 (no cone) in water.

Profiles were measured at d90/2, dmax, d90, d70, d50, and Rp + 2 cm depths for SSD 100 cm in water (Pinnacle requirement) and at 5 cm for 6 and 8 MeV and 10 cm for up to 15 MeV (Monaco requirement).

In‐air profiles were measured with the 25 × 25 cone at different source‐detector distance (SDDs) to calculate virtual SSD and sigma‐theta values for Pinnacle beam modeling. In‐air electron inplane and crossplane profiles for 8 × 8, 8 × 20, 8 × 40, and 40 × 40 fields without electron cone were measured at two different SSDs (e.g., 70 and 90 cm) for Monaco beam modeling. In‐air electron profiles with jaws in position as if cones were present were measured for Eclipse beam modeling.

Cone factors for all available cones including a 40 × 40 field without cone were measured in water for different SSDs (100 cm–120 cm). Output factors in air for 8 × 8, 8 × 20, 8 × 40, and 40 × 40 fields at two different SSDs (e.g., 70 and 90 cm) were measured for Monaco beam modeling.

### 2.D Beam modeling process

Pinnacle beam modeling can be done in‐house with the beam modeling tool that is available in the Pinnacle system. The Pinnacle beam modeling guide for linear accelerators with the Agility head is very detailed and helpful. The beam modeling tool allows the optimization of a large number of beam model parameters to match measured data as well as possible, but because of the complexity of this process, beam modeling in Pinnacle is time consuming and requires experience. Glenn et al. provide an overview of typical modeling parameters used for Pinnacle beam models of linear accelerators with the Agility head and Bedford et al. show that good results can be achieved even for complex VMAT plan calculations.^9^, ^10^

Eclipse beam modeling can also be done in‐house. When converting measured data in PTW’s default .mcc format to Eclipse compatible .w2CAD file format, it was determined that duplicate point data would prevent import of scanning data in the Beam Configuration workspace of Eclipse. Duplicate points were a result of processing scanning data inside the Mephysto software. Data were processed by applying smoothing profiles, then applying a CAX correction and symmetrizing profiles afterwards. This resulted in the duplicate data points, which were removed prior to import. This was accomplished by resampling processed data inside the Mephysto software. A simple modification in the Eclipse data import routine could possibly allow duplicate data entries to be ignored during data import. Once all measured data have been successfully imported, compared to Pinnacle very few parameters need to be manually manipulated in Eclipse beam modeling the MLC leaf gap parameter in Eclipse, which needs to be optimized for VMAT delivery. It is also important to know that customers may need to purchase an Elekta optimization license, even if they already have VMAT and IMRT optimization licenses for Varian linear accelerators.

Monaco beam modeling is done by Elekta, as Monaco does not have a beam modeling tool for customers. Scan data and forms need to be submitted online for this. Customers will receive a detailed beam modeling report once Elekta has finished the beam modeling for the linac. The beam model will be installed remotely and after validation needs to be approved by the on‐site medical physicist for clinical use.

### 2.E Beam model validation

Electron and photon beam models were validated based on the accuracy of model data agreement with measured input data and the correct use of absolute dose at the reference depth for each energy.

For further beam model validation, photon plans with open fields, wedged fields (Pinnacle and Monaco only), irregular shaped fields, IMRT fields, and clinical VMAT fields were calculated in all three TPS and measured with a MapCHECK device, Delta4, or Octavius device (Table 3). Measured dose planes were then compared to calculated dose planes.

**TABLE 3:** Evaluation tools.

Measurement device	Analysis software	Basic description
Seven29 array, PTW Octavius phantom, Freiburg, Germany	VeriSoft 5.1 (5.1.0.35)	729 vented plane‐parallel ionization chambers covering 27 cm × 27 cm area. Distance between the center of each chamber is 10 mm, with each chamber 5 mm × 5 mm × 5 mm. Surrounding material is PMMA
MapCHECK®, model 1175 Sun Nuclear, Melbourne, Florida	SNC Patient 6.6.0.32313	445 N‐type diodes, 0.8 mm × 0.8 mm active area of each diode, spacing 07.07 mm in central 10 cm × 10 cm area, spacing 14.14 mm in outer detectors in remaining 22 cm × 22 cm area
Delta^4^ diode array phantom, Scandidos, Uppsala, Sweden	Delta4® Version 2014 February	1069 p‐type silicon diodes in two orthogonal planes. Sensitive volume 0.78 mm^2^. Thickness is 0.05 mm. Spaced at 0.5 cm intervals in central 6 × 6 cm^2^ area and 1 cm intervals outside, covering 20 cm × 20 cm. Detector planes are in acrylic phantom

For clinical evaluation of the Pinnacle beam model (version 9.6), treatment plans were generated with open fields (square fields, rectangular fields, asymmetric, and irregular shaped fields) and wedged fields (5 × 5 to 20 × 20 field sizes; 15 to 60 degree wedge). In addition, four fields were selected of each, a clinical prostate and head and neck step and shoot IMRT plan. For all plans in Pinnacle, field gantry angles were set to 0° and fields were copied and calculated onto a water phantom for all available photon energies. These Pinnacle treatment plans were then exported from Pinnacle and imported into Eclipse and Monaco, where they were recalculated. Calculated dose planes from Pinnacle, Eclipse, and Monaco were then compared to the MapCHECK measurements. For these measurements the MapCHECK was setup with the SDD at 100 cm and the depth of calculation at 5 cm.

Additional validation plans were generated in Eclipse and then imported and recalculated in Monaco. These validation plans were provided to us by Varian (Palo Alto, CA) and represent their standard test plans, which are comparable to the standard set of plans as suggested in task group report 119 (TG‐119).^11^ The Varian test plans included symmetric and asymmetric rectangular fields, a C, E, O, and V shaped field, a slit field and a wide and narrow cross field, as well as two static head and neck and two static prostate fields. In addition, three step and shoot IMRT fields were evaluated. Calculated dose planes exported from Eclipse and Monaco were compared to MapCHECK measurements. For these measurements the MapCHECK was setup with the SDD at 100 cm and the depth of calculation at 5 cm.

To test the Elekta VMAT optimization in Eclipse, VMAT fields of treatment plans from Eclipse were measured with the Octavius device inside the Octavius hexagonal phantom for 10 MV photon beams, and with the Delta4 device for 6MV photon beams. For clinical evaluation of VMAT optimization and delivery, one VMAT H&N and one VMAT prostate patient plan were selected from our clinical Eclipse database. The original plans were delivered on either a Novalis Tx or Trilogy linear accelerator. The field geometry (arc angles, number of arcs/beams, etc.) was duplicated and the VMAT constraints used in the original plans were used for optimization of each plan with the Elekta beam model in Eclipse. Due to jaw tracking constraints it is not possible to change the energy of individual fields after copying the plan. It was necessary to delete the fields and recreate the fields with new energy definitions for each additional energy. The plans were copied and reoptimized using available energies 6 MV, 6 FFF, 10 MV, 10 FFF. Plans were evaluated for comparable dosimetric coverage and clinical endpoints. It is understood that it is not always clinically acceptable to use higher energy photons for all VMAT/IMRT plans, but for testing purposes plans were created with the 10 MV/10 FFF energies which met nearly all the clinical endpoints. Testing was not performed on 18 MV as it would not be used for IMRT or VMAT with the VersaHD linear accelerator.

## 3 RESULTS AND DISCUSSION

### 3.A MLC configuration

For Eclipse, measurements were performed with sweeping gap distances of 2, 4, 6, 10, 14, and 16 mm. It was determined that the plans which used gaps <5 mm could not be used to define the DLG value because the Agility MLC dynamic delivery requires a minimum leaf gap of 5 mm to prevent collision of leaf ends. Therefore, measurements of the 2 and 4 mm sweeping gaps artificially inflated the calculated DLG value to >1 mm. Sweeping gap distances used for determination of DLG value were 6, 10, 14, and 16 mm. For 6X‐FFF and 6 MV we lowered the measured DLG‐value to achieve better agreement between measurement and calculation of IMRT/VMAT QA plans (see Table 4). It is important to note that the DLG value has a huge impact on the passing rates of VMAT and sliding‐window IMRT plans. With the optimized DLG value, pass rates of our 6 MV and 6 FFF VMAT plans improved from a lower 70% pass rate to a pass rate in the higher 90% range. The optimized value of 0.3 mm for the 6X‐FFF and 6MV DLGs values agrees with the value of a publication by Zhang et al.^8^ Since measured DLG values had to be changed to achieve better agreement between measurements and calculations of IMRT/VMAT QA plans, considerable time and effort could be saved in future projects by solving the problem via numerical iterations starting with reference values from other successfully commissioned machines.

**TABLE 4:** MLC parameters for Eclipse model.

Energy	Transmission factor	Dosimetric leaf gap (cm) original → new
Dosimetric properties of the MLC		
6X	0.00216	0.0595 → 0.0300
10X	0.00217	0.0769
18X	0.00193	0.0676
6X‐FFF	0.00142	0.0515 → 0.0300
10X‐FFF	0.00115	0.0860

MLC transmission measurements were acquired using the same setup as the DLG measurements by taking the ratio of a MLC blocked field to the equivalent 10 × 10 cm^2^ open field for each energy. The values used in the MLC tab did not require additional changes based on the VMAT and IMRT measurement results.

For Monaco, the MLC values provided by Elekta were not modified or further optimized. In Pinnacle MLC offset values were optimized manually through a trial‐and‐error process to maximize MapCHECK QA passing rates.

### 3.B Verification of clinical Pinnacle plans

In order to recalculate the treatment plans imported from Pinnacle in Eclipse, a separate beam model had to be created that would allow the x‐jaws under the MLC banks to have positions other than 20 cm on each side. The x‐jaw positions on the Versa HD are not true positions, as the VersaHD does not have an x‐jaw. This problem arose due to a historical legacy of linear accelerators which do have x‐jaws. Pinnacle exported plans with a x‐jaw position even though the x‐jaw thickness in the Pinnacle beam model is 0 cm and there is no jaw to position. To allow a smooth import of VersaHD plans from other TPSs, Eclipse would have to allow to change the x‐jaw position for VersaHD linear accelerators automatically to 20 cm. The x‐jaw must be defined as nearly every TPS and record and verify system requires a numerical position for this accessory even if the accessory is not used with that system.

**FIG. 1:**
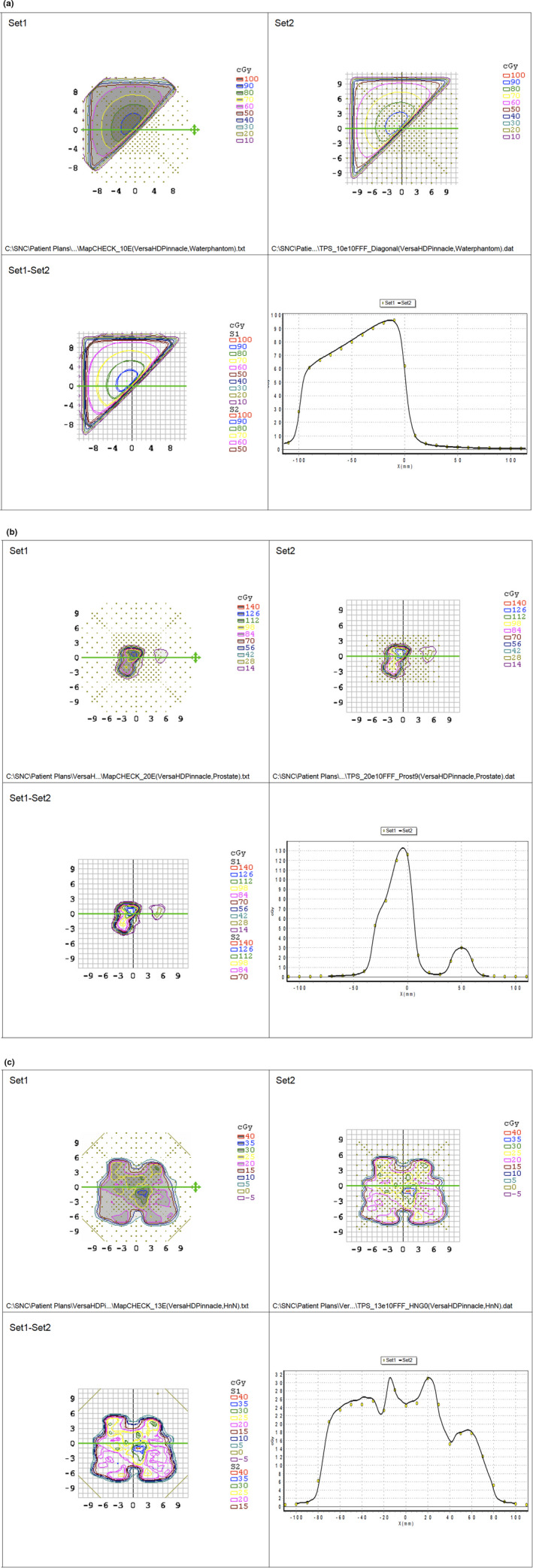
Examples of Pinnacle validation field measurements. The example on top (a) is a 20 × 20 field, which is blocked along the diagonal by the MLCs. The example below (b) is a highly modulated prostate IMRT field, and the example at the bottom (c) is a highly modulated head and neck IMRT field. Set 1 in the screenshots shows the dose distribution as measured with the Mapcheck device. Set 2 shows the dose distribution as calculated for the field by the treatment planning system. Set 1–Set 2 shows a comparison between Set 1 and Set 2. If a diode reading is below the defined tolerance, it is shown in blue; if a diode reading is above the defined tolerance, it is shown in red. The plot in the lower right corner of the screenshots shows a comparison between calculated (black line) and measured (yellow dots) dose along the green line shown in the Set 1–Set 2 window. Pass rates for this head and neck field are summarized in Table 5. The shown fields were all for the 10FFF beam and were analyzed with a 3%/3mm gamma, absolute dose, Van Dyk % pass criteria. The fields in (a) and (b) have a 100% pass rate, the field shown in (c) has a 98.2% pass rate.

Verification plans generated and calculated in Pinnacle were imported into Monaco and Eclipse, where they were recalculated. A comparison between Mapcheck measurements and dose distributions calculated for each field of these plans in Pinnacle, Monaco, and Eclipse shows that most fields pass with a 100% pass rate with the following pass criteria: 3%/3mm gamma, absolute dose, Van Dyk % difference. Figures 1(a) to 1(c) show examples. There were two exceptions: One of the irregular shaped field had a slightly lower pass rate in Pinnacle (98.2% for 6 MV and 6 FFF, 97.5% for 10 MV, 98.5% for 10 FFF, and 98.9% for 18 MV), compared to Eclipse (99.3% for 6FFF and 100% for all other) and Monaco (99.3% for 6FFF and 100% for all other). The second exception was a highly modulated head and neck IMRT field [Fig. 1(c)], which had slightly lower pass rates in all planning systems. Results are summarized in Table 5. As can be seen, however, all results would still be clinically acceptable.

**TABLE 5:** Passing rates for a highly modulated head and neck IMRT field (3%/3 mm gamma, absolute dose, Van Dyk % difference).

Energy	Pinnacle	Eclipse	Monaco
6 MV	98.2	96.7	98.2
10 MV	98.5	98.2	96.4
18 MV	90.9	95.4	94.4
6 FFF	98.9	98.2	98.9
10 FFF	98.2	96.4	97.8

### 3.C Verification of clinical Eclipse plans

As a result of evaluating the accuracy of the beam model (AAA.13.6.7) in Beam Configuration of Eclipse, the effective target spot size was set to 1 mm for 6 MV and 6 FFF energies to improve agreement in the model while for all other energies the effective spot size in the x‐ and y‐directions was 0 mm. Torsti et al.^6^ offer valuable guidance on the mechanics of tuning the source size. The paper also shows suitability of multiple source models for beam modeling across the entire model space with various field sizes, particularly small fields.

**FIG. 2:**
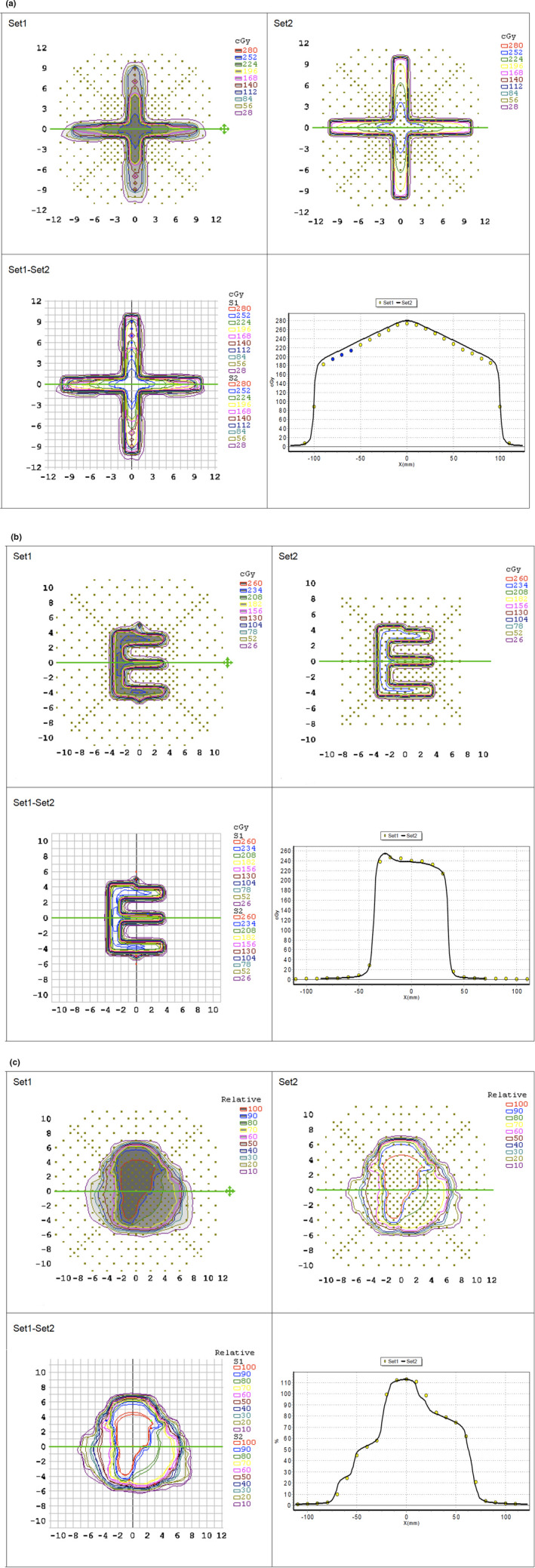
Examples of Eclipse validation field measurements. The example on top (a) is a 6FFF X‐shaped MLC field with a 94.1% pass rate for a 3%/3 mm gamma, absolute dose, Van Dyk % pass criteria. The example below is a 6FFF E‐shaped MLC field (b) with a 96.7% pass rate for a 2%/2 mm gamma, absolute dose, Van Dyk % pass criteria, and the example at the bottom is the 6FFF 4Composite MLC field (c) with a 94.8% pass rate for a 2%/2 mm gamma, absolute dose, Van Dyk % pass criteria.

Eclipse verification plan results from calculations in Eclipse and Monaco were found to be clinically acceptable (Table 6). Table 6 of the Eclipse results includes four plans which did not have a >90% passing rate at 3%/3 mm gamma index tolerance and two plans which did not have a >90% passing rate at 3%/3 mm gamma index tolerance from Monaco. All other plans which passed were evaluated at 2%/2 mm and 3%/3 mm and the tolerance required to achieve >90% passing rate is indicated. Plans which did not achieve passable results at 3%/3 mm criteria were investigated. Results were determined to be acceptable as the fields delivered were primarily narrow and the disagreement between planned dose distribution and measurement is attributable to the limited resolution of the MapCHECK detector. Examples of verification results can be found in Fig. 2. In general, pass rates between Eclipse and Monaco were found to be very comparable, with only slightly higher average pass rates for Monaco calculations.

**TABLE 6:** Eclipse verification field passing rates as calculated in Eclipse (bottom) and Monaco (top). Fields for which passing rates in Monaco were better compared to passing rates in Eclipse are highlighted in red.

	6 MV	10 MV	18 MV	6 FFF	10 FFF
	Gamma criteria/% points passing Gamma test				
Eclipse generated verification fields calculated in Monaco					
4 × 4	2/91.5	2/100		2/100	2/100
4 × 4_Asym	2/100	2/100		x	2/98.0
C	2/97.5	2/97.1		2/93	2/98.0
E	2/94.2	2/89.9		2/95.4	2/92.5
O	2/100	2/96.3		2/100	2/100
V	2/89.9	3/91.4		2/93.5	2/89.5
10 × 10 rect	3/99.1	2/94.1		2/100	2/100
BigCross	3/98.1	2/90.7		2/95.5	2/99.3
SmallCross	3/89.7	3/94.5		2/98.8	x
Slit	2/96.8	3/97.9		2/95.8	2/98.9
1HN	2/99..6	2/93.9.		2/100	2/98.8
2HN	2/99.6	2/93.2		2/100	2/100
1Pr	2/99.3	2/100		2/100	2/100
2Pr	2/100	2/96.8		2/100	2/98.7
3Composite	2/99.5	2/100		2/100	2/98.5
4Composite	2/99.6	2/100		2/99.1	2/100
5Composite	2/97.0	2/100		2/100	2/100

	6 MV	10 MV	18 MV	6 FFF	10 FFF
	Gamma criteria/% points passing Gamma test				
Eclipse generated verification fields calculated in Eclipse					
4 × 4	3/100	2/91.5	3/100	2/90.7	2/100
4 × 4_Asym	2/100	2/100	2/98.0	3/93.3	3/88.9
5 × 5_Rect	2/95.7	2/98.6	2/96.1	3/100	2/100
C	2/95.6	2/93.7	2/90.9	3/94.2	2/99.5
E	2/92.9	3/96.9	3/90.8	3/96.7	3/96.7
O	2/96.8	2/96.3	2/96.9	3/100	2/100
V	2/98.5	3/94.9	3/91.7	3/98.5	2/97.1
10 × 10 Rect	3/100	2/94.1	2/95.5	3/100	2/100
BigCross	3/88.8	3/100	3/100	3/94.1	2/98.7
SmallCross	3/100	3/96.4	3/85.8	3/98.8	3/90.0
Slit	2/100	2/93.7	2/97.9	3/92.6	3/81.1
1HN	2/96.9	2/95.4	2/97.0	2/98.4	2/100
2HN	2/98.1	2/95.0	2/95.9	2/97.6	2/99.6
1Pr	2/94.4	2/93.1	2/90.3	2/92.8	2/98.6
2Pr	2/98.0	2/93.6	2/92.1	2/96.0	2/97.3
3Composite	3/100	2/96.2	2/94.3	2/97.5	2/100
4Composite	2/94.6	2/99.2	2/97.5	2/94.8	2/100
5Composite	2/93.9	2/92.8	2/93.1	2/93.7	2/98.8

### 3.D Verification of Eclipse VMAT plans

No problems were found with optimizing Eclipse VMAT plans with the Elekta beam model. The optimization process was run through once for each plan to (a) reduce the time required to run multiple optimizations, (b) test the ability of the optimization system to produce clinically acceptable plans without having to modify the initial constraints, and (c) test the ability of the system to reproduce clinically acceptable plans for various energies without further modification.

All Eclipse 6 MV VMAT plans were calculated on the Delta4 phantom and measured with a clinically acceptable passing rate (see Table 7) using a gamma criteria no greater than 3%/3 mm with an acceptable threshold of 90%. All Eclipse 10 MV VMAT plans were delivered onto the Octavius phantom and evaluated using a gamma criteria of 2%/2 mm or 3%/3 mm with an acceptable threshold of 90%. (see Table 7). All results were found to be clinically acceptable as well.

**TABLE 7:** PTW Octavius and Delta4 VMAT QA results of Eclipse plans: Plans were analyzed using a gamma criteria of either 2%/2mm or 3%/3mm to achieve acceptable clinical results of ≥ 90% of points meeting this criteria.

	Gamma criteria	Passing rate (>90% OK)
VMAT prostate Delta4 QA results		
6MV	3%/3 mm	99.20% (DLG 0.03)
6FFF	3%/3 mm	98.30% (DLG 0.03)
VMAT HN Delta4 QA results		
6MV	3%/3 mm	97.40% (DLG 0.03)
6FFF	3%/3 mm	99.50% (DLG 0.03)
VMAT prostate Octavius QA results		
10MV	2%/2 mm	98.50%
10FFF	2%/2 mm	95.50%
VMAT HN Octavius QA results		
10MV	3%/3 mm	97.70%
10FFF	2%/2 mm	97.00%

## 4 CONCLUSION

In order to obtain an optimal clinical beam model for Monaco, Eclipse, and Pinnacle, a number of factors had to come together. First, proper measurement technique was vital to provide a full set of high‐quality beam data of vendor‐recommended profiles and factors to the fitting software of each planning system. The quality of these data will influence the ability of the modeling tools to achieve a good fit. Second, all fits were reviewed and simple test plans with point readouts were calculated. Third, verifications were done by means of point measurements. Finally, the volume array method of measuring validation plans was well characterized. This included verifying the phantom representation on which validation plans were computed has appropriate density as well as spatial dimensions. Dose and array calibrations were considered in relation to absolute point measurements. Furthermore, it was essential for the arrays to be optimally positioned on the table and to control all dosimetrically relevant errors during the validation measurements.^3^

The Eclipse version tested here was demonstrated to be capable of producing clinically acceptable treatment plans which are deliverable on a VersaHD linear accelerator and which are in dosimetric agreement with our measurements. Monaco and Pinnacle beam models also passed validation measurements in homogeneous materials for a large variety of different treatment fields. In the case of Eclipse the “dosimetric leaf gap” parameter was found to be critical for passing rates of VMAT plans. In the case of Pinnacle the MLC offset table needed to be optimized.

Each of these TPSs met the criteria to be used clinically in conjunction with Elekta linear accelerators. This shows, that a linear accelerator purchase decision does not need to drive or force a decision to switch to a different TPS, as this could be very disruptive and costly for a radiation oncology department. On the other hand, an existing TPS also does not need to drive or force the decision to purchase a linear accelerator from a specific vendor. This is important, as the decisions surrounding linear accelerator or TPS purchases are very complicated and not just limited to technical considerations. It can be of great advantage to have the option to operate in a mixed‐vendor environment.

There is a risk that the beam modeling process exhausts medical physicists before it has reached an optimal state. For this reason, vendors should further refine and automate their beam modeling process and tools. This would include fixing known inconsistencies as well as adding tools such as the ability to schedule time‐consuming calculations to run without oversight during nonclinical hours. A data validation test suite could be built to detect common measurement and modeling errors as well as iterate numerically against measured plans to tune the parameters. Medical physicists should search for publications of typical beam model parameters of TPS beam model for their linear accelerator, such as the publication by Bedford et al. or the recently published paper by Glenn et al.^10^, ^12^ Such reference data can help spot potentially suspicious beam model parameters.

## AUTHOR CONTRIBUTIONS

All authors meet the following requirements: They made substantial contributions to the conception and design of the work; or the acquisition, analysis, or interpretation of data for the work; Helped draft the work and revising it critically for important intellectual content; Gave final approval of the version to be published; Agree to be accountable for all aspects of the work in ensuring that questions related to the accuracy or integrity of any part of the work are appropriately investigated and resolved.

## CONFLICT OF INTEREST

The authors have no conflict of interest.

5

**FIG. 3:**
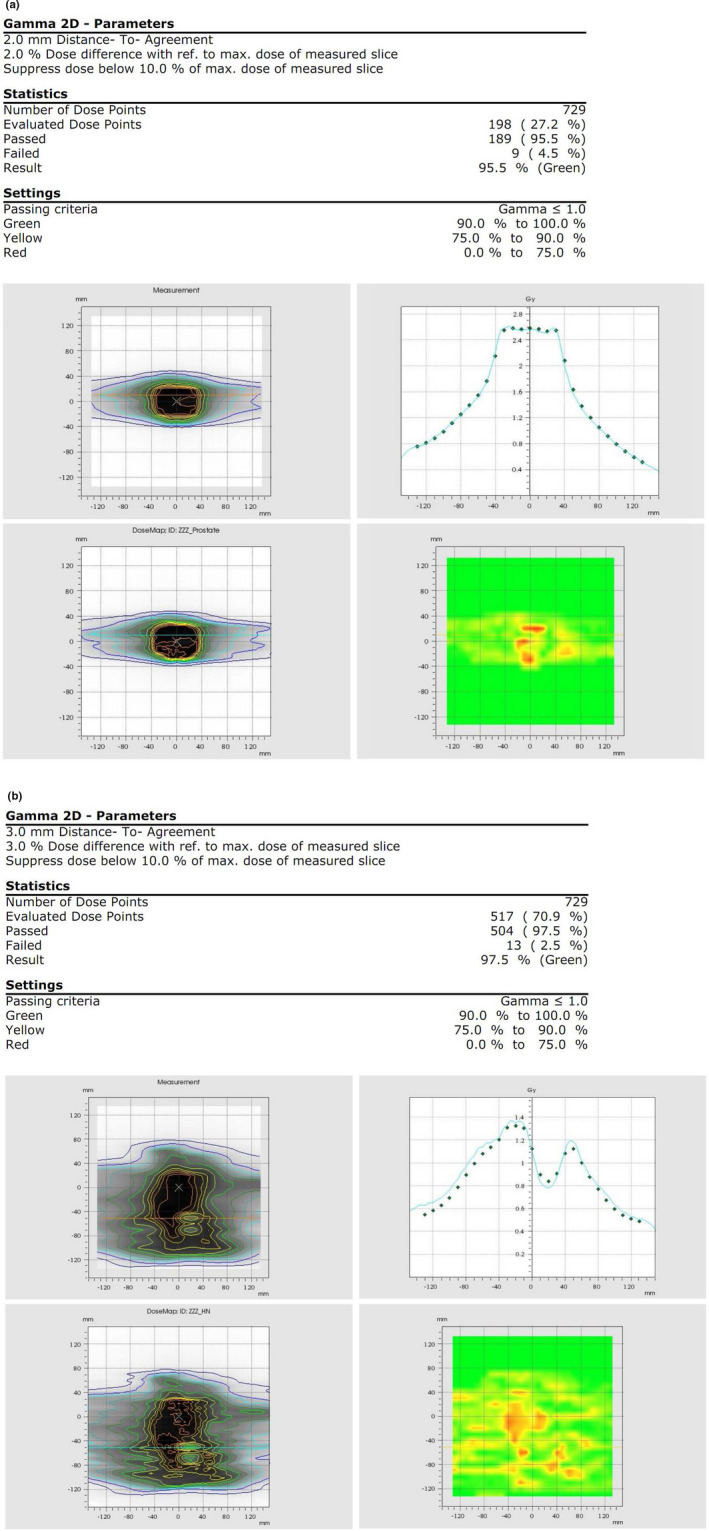
Clinical VMAT field QA measurements – 10 FFF prostate (a, top) and 6 FFF head and neck (b, bottom) case.
